# Novel variants in helicase for meiosis 1 lead to male infertility due to non-obstructive azoospermia

**DOI:** 10.1186/s12958-021-00815-z

**Published:** 2021-08-24

**Authors:** Dongdong Tang, Mingrong Lv, Yang Gao, Huiru Cheng, Kuokuo Li, Chuan Xu, Hao Geng, Guanjian Li, Qunshan Shen, Chao Wang, Xiaojin He, Yunxia Cao

**Affiliations:** 1 Reproductive Medicine Center, Department of Obstetrics and Gynecology, the First Affiliated Hospital of Anhui Medical University, No 218 Jixi Road, Hefei, 230022 Anhui China; 2 NHC Key Laboratory of Study On Abnormal Gametes and Reproductive Tract, Anhui Medical University, No 81 Meishan Road, Hefei, 230032 Anhui China; 3 Key Laboratory of Population Health Across Life Cycle, Anhui Medical University, Ministry of Education of the People’s Republic of China, No 81 Meishan Road, Hefei, 230032 Anhui China

**Keywords:** Male infertility, Non-obstructive azoospermia, *HFM1*, microTESE

## Abstract

**Background:**

Non-obstructive azoospermia (NOA) is the most severe form of male infertility; more than half of the NOA patients are idiopathic. Although many NOA risk genes have been detected, the genetic factors for NOA in majority of the patients are unknown. In addition, it is difficult to retrieve sperm from these patients despite using the microsurgical testicular sperm extraction (microTESE) method. Therefore, we conducted this genetic study to identify the potential genetic factors responsible for NOA and investigate the sperm retrieval rate of microTESE for genetically deficient NOA patients.

**Methods:**

Semen analyses, sex hormone testing, and testicular biopsy were performed to categorize the patients with NOA. The chromosome karyotypes and Y chromosome microdeletion analyses were used to exclude general genetic factors. Whole exome sequencing and Sanger sequencing were performed to identify potential genetic variants in 51 patients with NOA. Hematoxylin and eosin staining (H&E) and anti-phosphorylated H2AX were used to assess the histopathology of spermatogenesis. Quantitative real time-polymerase chain reaction, western blotting, and immunofluorescence were performed to verify the effects of gene variation on expression.

**Results:**

We performed whole exome sequencing in 51 NOA patients and identified homozygous helicase for meiosis 1(*HFM1*) variants (NM_001017975: c.3490C > T: p.Q1164X; c.3470G > A: p.C1157Y) in two patients (3.9%, 2/51). Histopathology of the testis showed that spermatogenesis was completely blocked at metaphase in these two patients carrying the *HFM1* homozygous variants. In comparison with unaffected controls, we found a significant reduction in the levels of HFM1 mRNA and protein expression in the testicular tissues from these two patients. The patients were also subjected to microTESE treatment, but the sperms could not be retrieved.

**Conclusions:**

This study identified novel homozygous variants of *HFM1* that are responsible for spermatogenic failure and NOA, and microTESE did not aid in retrieving sperms from these patients.

**Supplementary Information:**

The online version contains supplementary material available at 10.1186/s12958-021-00815-z.

## Introduction

Azoospermia is a medical condition characterized by the absence of sperms in the ejaculated semen. Azoospermia is the most severe form of male infertility and accounts for approximately 1% of the total male population and 10% of the infertile males globally [1, 2]. Based on pathological features, azoospermia can be classified into obstructive azoospermia (OA) and non-obstructive azoospermia (NOA). With the development of assisted reproductive technology, techniques such as intracytoplasmic sperm injection have made it possible for most patients with OA to conceive their own offspring [3, 4]. However, it is a challenge to retrieve sperms from patients with NOA, even by microsurgical testicular sperm extraction (microTESE) [5, 6]. Therefore, it is important to investigate the etiology and pathogenesis of NOA, which will facilitate the development of a targeted therapy.

It has been reported that some factors, such as abnormal chromosomes, Y-chromosomal microdeletion, and cryptorchidism, can cause NOA. However, a majority of the NOA cases remain idiopathic, and no medical etiologies have been identified [4]. In recent years, it has been reported that several genes, such as *TEX11* (OMIM: 300,311), *TEX14* (OMIM: 605,792), *FANCM* (OMIM: 609,644), *SPINK2* (OMIM: 605,753), *MEIOB* (OMIM: 617,670), and *STAG3* (OMIM: 608,489), are associated with spermatogenic defects in patients with NOA [7–13]. Although the identified genes only account for a small proportion of NOA cases, they strengthen our understanding of the causes of NOA.

Helicase for meiosis 1 (HFM1) plays an important role in crossing over and synapsis during meiosis [14]. Previous studies have reported that variations in human *HFM1* are related to female premature ovarian failure (POF) and male NOA or severe oligozoospermia [15, 16]. The phenotypes of POF and NOA are also found in *Hfm1*^−/−^ female and male mice, respectively [17]. In this study, we identified two novel homozygous variants of *HFM1* in two patients with NOA, which strengthens the clinical significance of *HFM1* for NOA phenotype. Additionally, we also investigated the results of microTESE in patients with *HFM1* variants for the first time, which may provide a clinical reference that microTESE is not the ideal extraction method for such patients.

## Methods

### Subjects

A cohort of 51 Chinese men with idiopathic NOA were enrolled from the First Affiliated Hospital of Anhui Medical University. The ejaculated semen and urine were centrifuged and analyzed, together with some other tests were performed, included the determination of somatic karyotypes, screening of Y chromosome microdeletions, sex hormone testing, subsequent testicular biopsy, and testicular pathological analysis to identify the NOA phenotype in these patients. Participants with a history of cryptorchidism, testicular torsion, epididymitis, epididymo-orchitis, mumps orchitis and/or ascending sexually transmitted infections were not included in the study because of the causative relationships of such pathologies with obstructive/non-obstructive azoospermia [18–20]. The patients with abnormal somatic karyotypes and Y chromosome microdeletions were also excluded from this study.

### Ethical approval

All participants signed a written informed consent to participate in the study. The study was approved by the review board committee of the First Affiliated Hospital of Anhui Medical University, and it was conducted in accordance with the Declaration of Helsinki.

### Whole exome sequencing (WES), Sanger sequencing, and bioinformatics analysis

DNA was extracted from the whole peripheral blood of the patients. WES, Sanger sequencing, and bioinformatics analysis were performed according to previous studies [18, 19]. In summary, we annotated variants using allele frequency databases (1000G, EXAC, gnomAD), deleterious prediction tools (SIFT, PolyPhen-2, Mutation Taster, and CADD), and Genotype-Tissue Expression database by ANNOVAR, VarCards and dbNSFP [21–23]. The variants with allele frequencies > 0.05 were excluded. We retained deleterious missense variants and loss-of-function variants including splicing (≤ 2 bp), stop-gain, stop-loss, and frameshift variants. The variant that was predicted to be deleterious by three of the four tools, namely, SIFT, PolyPhen-2, Mutation Taster, and CADD (score > 20), was defined as a deleterious variant. The pathogenic variants expressing testis-specific genes were used for further analysis. Testis-specific expression genes were defined as those genes having an average expression value ≥ 5 reads per kilobase per million mapped reads in human testis and a value that is two folds higher than the average expression value in other tissues based on the Genotype-Tissue Expression data. For patients from consanguineous families, we focused on autosomal homozygous variants or X-linked variants. Finally, we further assessed the potential function and phenotype of the selected gene using Gene Ontology database, Online Mendelian Inheritance in Man database, Model Organism database, and PubMed based literature review. Sanger sequencing was performed to identify parental origins of pathogenic variants. The primers used are listed in Supplementary Table 2.

### Hematoxylin and eosin (H&E) and immunofluorescence assay

H&E staining and immunofluorescence assay were performed as previously described [24, 25]. Histopathological assessment of spermatogenesis was performed by H&E staining. The location of candidate gene expression in seminiferous tubules was determined by immunofluorescence. The following antibodies were used: mouse monoclonal antibody againstγH2AX [(1:1000; Millipore, 05–636) to detect meiotic double-strand breaks (DSBs) and the XY body in pachytene nuclei and apoptotic metaphases in case of pan-chromosomal signal, rabbit polyclonal anti-H3Ser10ph (1:1,000; 06–570, Millipore), and peanut agglutinin (PNA) conjugated to rhodamine (1:500; Vector laboratories, RL-1072). The Probable ATP-dependent DNA helicase HFM1 antibody was used as the primary anti-HFM1 antibody (1:50; Abbexa, abx124216).

### Quantitative real-time PCR (qPCR) and western blotting

The mRNA and protein expression levels of the candidate gene were determined by qPCR and western blotting, respectively, according to previously described procedures [25, 26]. The primary anti-HFM1 antibody (Abbexa) was used for both immunofluorescence (1:50) and western blotting (1:1000); the PCR primers used are listed in Supplementary Table 3.

### MicroTESE

As no sperm was detected in routine testicular biopsy, microTESE was performed to retrieve testicular sperms, according to a previous study [27].

## Results

### Homozygous variants in HFM1 were identified in two Chinese men with NOA

**Fig. 1:**
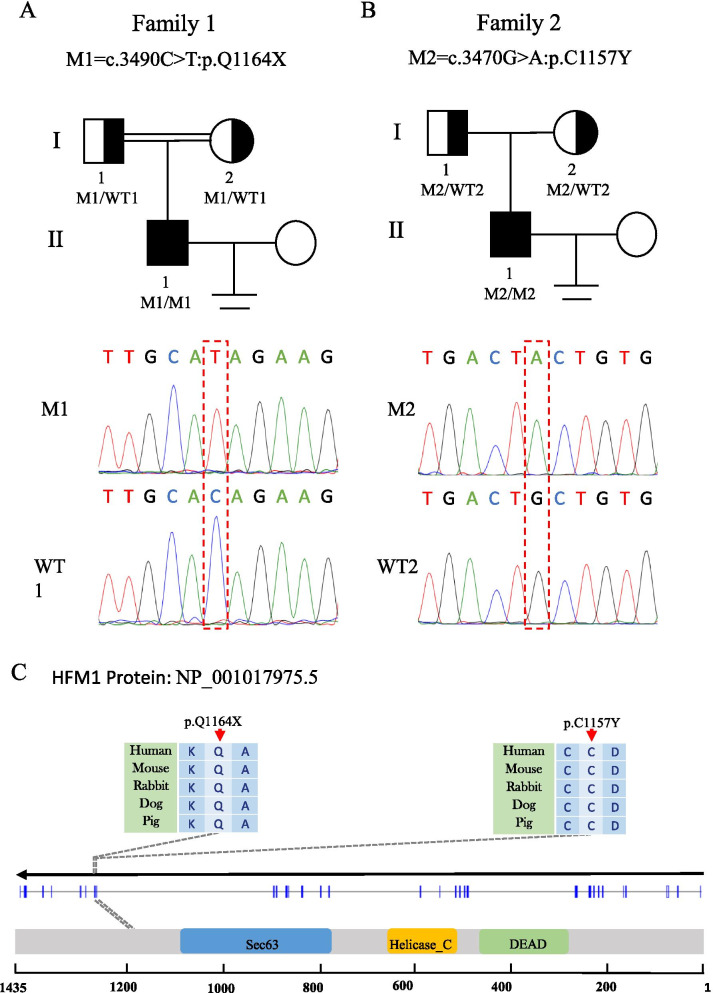
Identification of bi-allelic *HFM1* variants in two Chinese azoospermic men. **A-B** The two families affected by the variants in *HFM1*. The red frames indicate mutated positions in the Sanger sequencing results. **C** The affected amino acid residues of *HFM1* are conserved among species. The red arrows indicate the locations of *HFM1* variants occurring in the domains of HFM1 protein. M, *HFM1* mutation; WT, wild type

We performed bioinformatics analysis of the WES data of one patient with NOA from a consanguineous family (F1 II-1) and found a homozygous *HFM1* loss-of-function variant (NM_001017975: c.3490C > T: p.Q1164X), which was absent in our 500 in-house Chinese unrelated cohort controls in the general population. For other variants that passed the filter threshold, no potential NOA related variants in F1 II-1 were observed (Supplementary Table 1). Sanger sequencing confirmed that the patient was homozygous for the variant and his parents were identified as heterozygous carriers (Fig. 1A). In addition, we also found a pathogenic homozygous missense variant of the *HFM1* gene (NM_001017975: c.3470G > A: p.C1157Y) in one of the remaining 50 patients from non-consanguineous families (F2 II-1) (Fig. 1B). This variant was rare in our 500 in-house Chinese unrelated cohort controls in the general population. Moreover, this variant was defined as deleterious by the four prediction tools (Table 1). Similarly, no potential NOA related variants in F2 II-1 were observed (Supplementary Table 1).

**Table 1:** Clinical features and genetic information of *HFM1* mutations in F1 II-1 and F2 II-1

Individuals	F1 II-1	F2 II-1
Clinical features		
Age	28	31
Secondary sexual characteristics	Normal	Normal
Testicular volume(Left/Right, ml)	12/12	10/10
Somatic karyotype	46,XY	46,XY
Y Chromosome microdeletions	No	No
Sex hormone		
Follicle-stimulating hormone(IU/L)	14.75	25.79
Luteinizing hormone(IU/L)	7.53	15.10
Testosterone(nmol/L)	11.04	12.86
Estradiol(pmol/L)	209.00	109.00
Prolactin(ng/ml)	13.92	6.34
Information of *HFM1* mutations		
cDNA mutation	c.3490C > T	c.3470G > A
Mutation type	Stopgain	Missense
Protein alteration	p.Q1164X	p.C1157Y
Allele frequency in human population		
CHN500	0	0.02
1KGP	0	0.005
ExAC_all	0	0.002
gnomAD	0	0.002
Functional prediction		
SIFT	N/A	Damaging
PolyPhen-2	N/A	Probably Damaging
MutationTaster	Disease_causing Automatic	Disease_causing
CADD	45	29.7

RefSeq accession number of *HFM1* is NM_001017975.5

*Abbreviations*: *CHN500* 500 unrelated controls in Chinese, *1KGP* 1000 Genomes Project, *ExAc_all* all the data of Exome Aggregation Consortium, *gnomAD* the Genome Aggregation Database, *N/A* Not applicable

*HFM1* (the human homologue of yeast Mer3), a meiotic gene, is located on human chromosome 1p22.2 and contains 39 exons encoding a predicted 1435-amino acid protein, highly expressed in the testis and ovaries. HFM1 protein is mainly composed of helicase ATP-binding domain, helicase C-terminal domain, and SEC63 domain. The two homozygous variations found in this study were located in the zinc finger motif of HFM1 protein. F1 II-1, harboring the stop-gain variant p.Q1164X, introduces a premature stop codon and thus is expected to produce either no protein or truncated non-functional proteins, while the variant p.C1157Y might lead to a change at splice donor site 3 bp downstream in exon 31. These two mutated residues are conserved in many organisms and may probably lead to HFM1 protein dysfunction (Fig. 1C).

### Meiotic metaphase arrest in the testis of patients

**Fig. 2:**
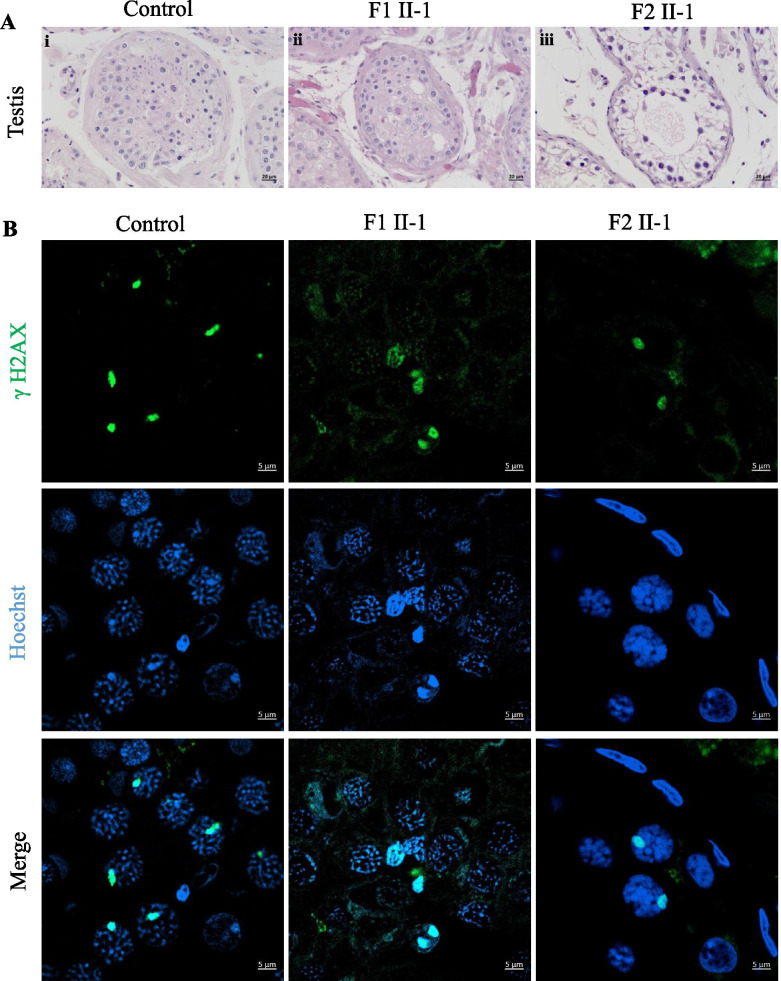
Investigation of patients harboring the *HFM1* variants and the controls. **A** Testicular histological sections from an OA patient (control-i) and patients harboring the *HFM1* variants (F1 II-1-i, F2 II-1-ii) were stained with hematoxylin and eosin (H&E). Scale bar represents 20 μm. **B** Immunofluorescent staining of histological sections from the testis biopsy of the control and patients carrying the *HFM1* variants usingγH2AX (green, marker for DSBs and XY body) and Hoechst (blue). Many aberrant γH2AX spots in samples of F1 II-1 were observed, such as small patches of γH2AX staining or two (or more) γH2AX-positive XY body–like structures. Extremely reduced XY body positive tubules were observed in samples of F2 II-1, indicating that the cells rarely reach the pachytene stage. Scale bar represents 5 µm

**Fig. 3:**
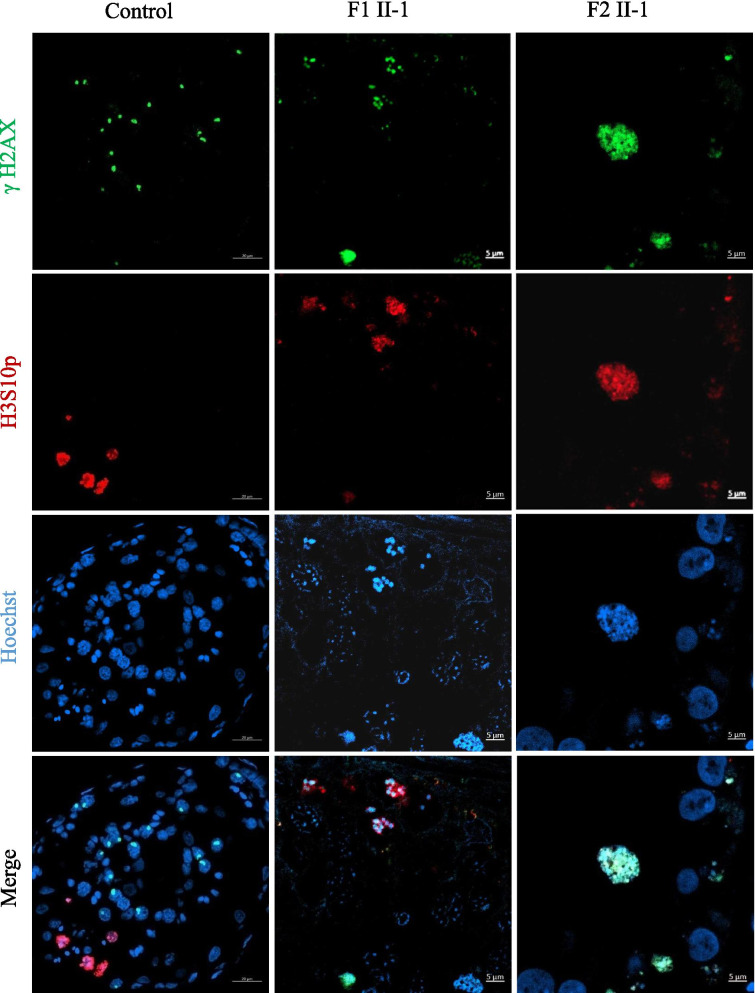
Immunofluorescent staining of histological sections from the testis biopsy of the patient carrying the *HFM1* variants using γH2AX (green), H3S10p (red), and Hoechst (blue). Scale bar represents 5 μm. A high density of metaphases was observed and almost all the metaphases were apoptotic. The organization of the apoptotic metaphases appeared to be more dispersed in samples of F1 II-1. In F2 II-1 patient rare metaphases were observed and all the metaphases displayed an aberrant pattern of γH2AX spots whereby γH2AX patches covered the entire nucleus

**Fig. 4:**
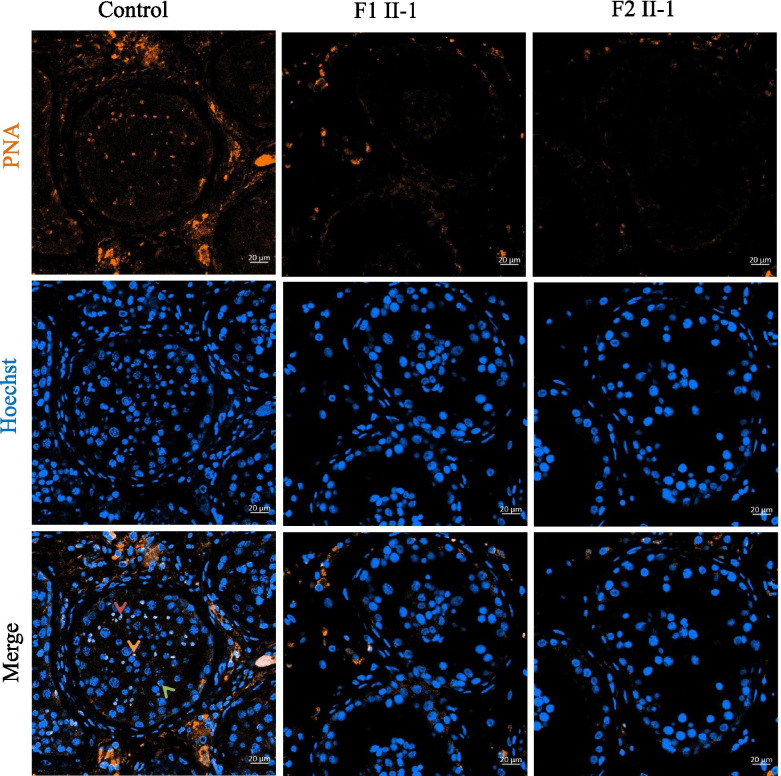
Immunofluorescent staining of histological sections from the testis biopsy of the patient carrying the *HFM1* variants using peanut agglutinin (PNA, brown) conjugated to rhodamine to locate the acrosome of spermatid. Compared with the control, the spermatid was absent in testis from men harboring biallelic *HFM1* variants, indicating a premetaphase arrest. Hoechst (blue) was stained as a nuclear marker. Scale bar represents 20 μm

To determine the potential effects of the *HFM1* homozygous variants on human spermatogenesis, testicular histological tissues were collected from F1 II-1 and F2 II-1 and analyzed using H&E staining. Compared to the normal control, the seminiferous tubules from the testis of patients carrying the *HFM1* variants showed reduced diameters and the absence of spermatid (Fig. 2A). To further assess meiotic progression of testis in patients carrying the homozygous variants, we stained the histological sections using anti-phosphorylated H2AX (γH2AX) alone or with a combination of γH2AX and anti-phosphorylated H3 (H3Ser10ph) antibodies. As a marker of meiotic DSBs and the XY body, γH2AX was used to verify the meiotic phase, the progression of DSB repair, and the formation of XY body. We found that a portion of the tubule section was marked by the presence of multiple γH2AX patches which indicated the stage of spermatocytes, including leptotene and zygotene (Fig. 2B). Moreover, we observed two (or more) γH2AX-positive XY body like structures in the testis of the F1 II-1 patient and extremely reduced XY body positive tubules in the testis of the F2 II-1 patient (Fig. 2B). These results indicate that the cells of the testis rarely reach the pachytene stage in the *HFM1* variants. In addition, H3Ser10ph immunostaining was used to identify M-phase cells; the metaphases also displayed intense γH2AX signals along the condensed chromosomes. Since this type of pan-chromosomal γH2AX staining has been described as a hallmark of cells entering apoptosis, for *HFM1* variants subjects, most of the metaphase cells were apoptotic. The organization of the apoptotic metaphases appeared to be more dispersed in the testis of the F1 II-1 patient than that in the F2 II-1 patient. In F2 II-1 patient, rare metaphases were observed and all the metaphases displayed an aberrant pattern of γH2AX spots, where the γH2AX patches covered the entire nucleus (Fig. 3). Moreover, round (green arrow), elongating (red arrow), and condensing spermatids (brown arrow) could be observed in the control sample using the combined label of acrosome antibody and Hoechst. However, spermatid was absent in the testis of men harboring homozygous *HFM1* variants (Fig. 4). All these findings suggested that the deficiency of *HFM1* largely resulted in the blocking of spermatogenesis at the pachytene stage; some spermatocytes that developed further exhibited dramatic apoptotic metaphases and complete metaphase arrest.

After an exhaustive understanding of microTESE, two patients consented to undergo the procedure. However, using this technique, sperms were not found in any of the multiple testis fragments from the two patients carrying the homozygous variants, although spermatocytes were observed. Thus, microTESE further confirmed our histological observations.

## *HFM1* expression were reduced in the testicular tissues of patients

**Fig. 5:**
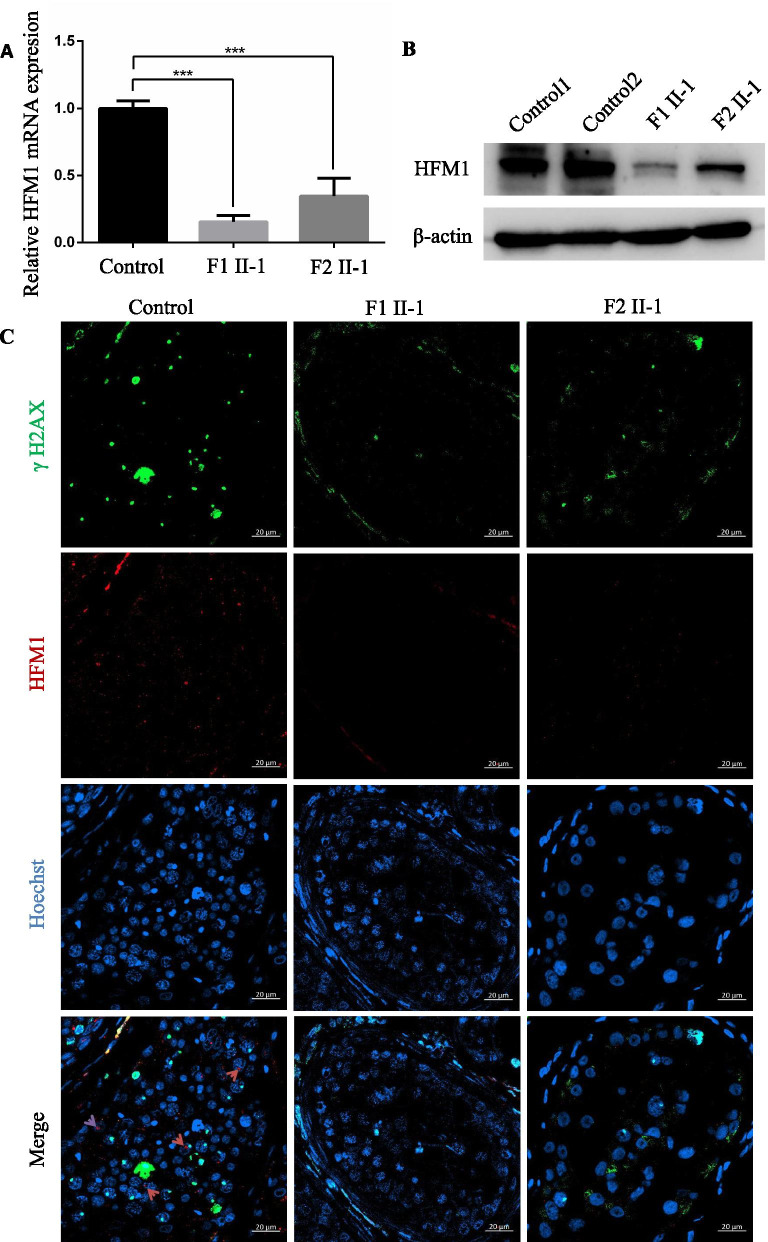
Expression and location analysis of HFM1 protein in the testis from the control patient and men harboring bi-allelic *HFM1* variants. **A** qPCR analysis indicated that the abundance of *HFM1* mRNA decreased significantly in the testis of men harboring homozygous *HFM1* variants when compared to that of a normal control male. Data represents the means ± SEM. (standard error of measurement) of three independent experiments. Two-tailed Student’s paired or unpaired t tests were used as appropriate (*** *P* < 0.001). **B** Western blotting assay revealed that HFM1 protein levels reduced significantly in the testis from men harboring *HFM1* mutations. β-actin was used as a loading control. **C** HFM1 localization in the testis from a control individual and men harboring bi-allelic *HFM1* variants. HFM1 immunostaining (red) was primarily concentrated in cytoplasm of spermatogonia and spermatocytes in seminiferous tubules in normal control. The immunostaining was decreased in the testicular tissues of F1 II-1 and F2 II-1. Hoechst (blue) was stained as a nuclear marker. The γH2AX (green) was stained as a marker of spermatocyte. Scale bars: 20 μm

To investigate the pathogenicity of the *HFM1* variants identified in this study, we examined the expression levels of *HFM1* mRNA and protein in the testis from the patients harboring the homozygous *HFM1* variants and the controls. QPCR results suggested that the abundance of *HFM1* mRNA was significantly reduced in the testis of men harboring the homozygous *HFM1* variants (Fig. 5A). Consistently, the expression of HFM1 protein was also dramatically reduced in the testicular tissues of F1 II-1 and F2 II-1, compared to the control (Fig. 5B).

Furthermore, we performed immunofluorescence analysis of HFM1 expression in the testicular tissues of affected individuals in comparison with OA control. Immunostaining signal of the testicular tissues from OA patient revealed that HFM1 was mainly concentrated in the cytoplasm of spermatogonia and spermatocytes in the seminiferous tubules. In contrast, HFM1 signal was significantly reduced in the testicular tissues of both F1 II-1 and F2 II-1 (Fig. 5C).

## Discussion

In this study, two homozygous *HFM1* variants were identified as the genetic factor responsible for the impairment of spermatogenesis in two patients with NOA. The deficiency of *HFM1* resulted in spermatogenesis being blocked at the pachytene stage. However, some spermatocytes apparently developed further, and a dramatic increase in the number of apoptotic metaphases indicated a complete metaphase arrest. Additionally, HFM1 protein was mainly concentrated in the cytoplasm of spermatogonia and spermatocytes in the seminiferous tubules of the control (OA) patient. The expression levels of HFM1 mRNA and protein significantly decreased in men carrying the homozygous *HFM1* variants. Moreover, no sperms were recovered by microTESE from *HFM1* mutated subjects. These results indicate that *HFM1* variants are novel causative variants of NOA in humans.

*HFM1* (also known as Mer3 in yeast) is highly expressed in tissues of the testis and ovaries, contains 39 exons, and encodes a predicted 1435-amino acid protein, which is required in many organisms for crossover formation and the complete synapsis of homologous chromosomes during meiosis [14, 17]. Two variants found in this study were located in the zinc finger motif of HFM1 protein; F1 II-1, harboring a stop-gain variant p.Q1164X, introduced a premature stop codon resulting in the absence of protein expression, while the homozygous missense variant p.C1157Y, reported previously by Zhang et al*.*, might lead to a change at splice donor site 3 bp downstream in exon 31 resulting in a significant decrease in the expression of HFM1 protein [16]. These two mutated residues are conserved in many organisms and probably lead to HFM1 protein dysfunction, thus blocking spermatogenesis at the pachytene stage. In addition, Zhang et al*.* found that *HFM1* variations were associated with idiopathic azoospermia or severe oligozoospermia in Chinese men, which further confirmed our results [16].

Similarly, knockout of *Hfm1* in male mice showed that spermatogenesis is blocked at diakinesis of meiosis I while apoptosis of spermatocytes at diakinesis is common in the seminiferous tubules of *Hfm1*^*−/−*^ mice. Majority of the initial recombination events (homologous recognition, pairing, and initial synapsis) in *Hfm1*^*−/−*^ spermatocytes were normal compared to that in wild-type mice. In addition, synapsis was incomplete for most of the chromosomes in *Hfm1*^*−/−*^ spermatocytes, suggesting that HFM1 participates in a major crossover pathway [17]. However, these results were different from those in budding yeast or *C. cinereus*, where Mer3 deletion results in mid prophase I arrest, or in *S. macrospora*, where Mer3 mutants show a delay in the leptotene-zygotene transition [28–30]. These differences among species indicate that the biological functions that require HFM1/Mer3 and/or the responses to its absence have diverged along with other features of meiotic chromosome metabolism in these organisms.

Furthermore, *Hfm1*^*−/−*^ adult female mice presented a significant reduction in ovary size, reduced number of follicles, and increase in stromal cells and corpora lutea. The phenotypes were similar to those observed for primary ovarian insufficiency [15, 31, 32]. Hence *HFM1* is also known as POF9.

No sperms were observed in routine testicular biopsy of these two patients. Considering the high sperm retrieval rate by microTESE in NOA patients [33, 34], F1 II-1 and F2 II-1 consented to undergo the procedure. However, no sperms were retrieved from either of the patients. This is the first study to report the outcomes of microTESE in NOA patients with variations in *HFM1*. Although only two patients were enrolled, it may provide a clinical reference that microTESE is not beneficial for these patients.

However, this study had a few limitations. Firstly, since sequencing of *HFM1* was not performed in healthy fertile males, we cannot comment on the variant frequency of the gene in this population. Secondly, the chromosome spread experiment, which can detect possible defects during synapsis and recombination, was not performed due to shortage of testicular sample. Thirdly, only two cases with homozygous variants in *HFM1* underwent microTESE, further research is needed to verify the chances of successful sperm retrieval by microTESE in NOA patients with bi-allelic *HFM1* variants.

## Conclusions

Our results provide further evidence that *HFM1* is a candidate gene responsible for NOA in humans, and that homozygous variants in *HFM1* can cause autosomal recessive male infertility due to NOA. In addition, it is likely that microTESE cannot be used for sperm retrieval in these patients.

## Supplementary Information

**Additional file 1: Table S1.** Predicted as pathogenic variants indetified in two patients carrying HFM1 variants

**Additional file 2: Supplementary Table 2.** Primers used for verification of HFM1 variants.

**Additional file 3: Supplementary Table 3.** Primers used for QRT-PCR assay of HFM1 and β-actin.

## Data Availability

The datasets used and/or analyzed during the current study are available from the corresponding author on reasonable request.

## Abbreviations

NOA: Non-obstructive azoospermia
microTESE: Microsurgical testicular sperm extraction
WES: Whole exome sequencing
H&E: Hematoxylin and eosin staining
OA: Obstructive azoospermia
HFM1: Helicase for meiosis 1
POF: Premature ovarian failure
qPCR: Quantitative real-time PCR
ACMG: American College of Medical Genetics and Genomics
SIFT: Sorting Intolerant From Tolerant
