# Supplementary material for: Novel variants in helicase for meiosis 1 lead to male infertility due to non-obstructive azoospermia
Source: Reprod Biol Endocrinol. 2021 Aug 24;19:129. doi: 10.1186/s12958-021-00815-z (PMC8383409; doi:10.1186/s12958-021-00815-z)
Supplement: Supplementary file 2 — Additional file 2: Supplementary Table 2. Primers used for verification of HFM1 variants. [file 12958_2021_815_MOESM2_ESM.docx]

**Supplementary Table 2. Primers used for verification of *HFM1* variants.**

|  | **Primer Names** | **Primer Sequences (5'-3')** |
| --- | --- | --- |
| **F1 II-**1 | M1-F | TCTCTCTGTGGGCAACATGAAAA |
|  | M1-R | CACTGTGGATACCTAATTGACAGACAG |
| **F2 II-1** | M2-F | GTATTGTCTGAAAGGAAGGAAACTGG |
|  | M2-R | TTTCATGTTGCCCACAGAGAGAA |
