# Supplementary material for: Novel variants in helicase for meiosis 1 lead to male infertility due to non-obstructive azoospermia
Source: Reprod Biol Endocrinol. 2021 Aug 24;19:129. doi: 10.1186/s12958-021-00815-z (PMC8383409; doi:10.1186/s12958-021-00815-z)
Supplement: Supplementary file 3 — Additional file 3: Supplementary Table 3. Primers used for QRT-PCR assay of HFM1 and β-actin. [file 12958_2021_815_MOESM3_ESM.docx]

| **Supplementary table 3. Primers used for QRT-PCR assay of HFM1 and β-actin.** | | | |
| --- | --- | --- | --- |
|  | **Primer Names** | **Primer Sequences (5'-3')** | **Tm** |
| ***HFM1*** | Forward | ATGAGAGCCATAGACCAGTGAA | 60.3 |
|  | Reverse | ATGAGAGCCATAGACCAGTGAA | 61.3 |
| ***β-actin*** | Forward | CATGTACGTTGCTATCCAGGC | 60.8 |
|  | Reverse | CTCCTTAATGTCACGCACGAT | 60.2 |
